# Data-driven Modeling of Long-term CD4 Cell Recovery Trajectories Under Modern Antiretroviral Therapy in People Living With HIV

**DOI:** 10.1093/ofid/ofag181

**Published:** 2026-03-27

**Authors:** You Ge, Chunqin Bai, Anni Liu, Zheng Qian, Ziyao Liu, Chenyu Ma, Jinjin Yang, Zhixiang Dai, Kai Wang, YuanYuan Xu, Hongxia Wei, Zhengping Zhu, Zhihang Peng, Zhiliang Hu

**Affiliations:** Department of Infectious Diseases, Nanjing Public Health Medical Center, the Second Hospital of Nanjing, Nanjing University of Chinese Medicine, Nanjing, China; Department of Infectious Diseases, Nanjing Public Health Medical Center, the Second Hospital of Nanjing, Nanjing University of Chinese Medicine, Nanjing, China; Department of Infectious Diseases, Nanjing Public Health Medical Center, the Second Hospital of Nanjing, Nanjing University of Chinese Medicine, Nanjing, China; Department of Infectious Diseases, Nanjing Public Health Medical Center, the Second Hospital of Nanjing, Nanjing University of Chinese Medicine, Nanjing, China; School of Public Health, Nanjing Medical University, Nanjing, China; School of Public Health, Nanjing Medical University, Nanjing, China; School of Public Health, Nanjing Medical University, Nanjing, China; School of Public Health, Nanjing Medical University, Nanjing, China; School of Public Health, Nanjing Medical University, Nanjing, China; Department of AIDS/STD Control and Prevention, Nanjing Municipal Central for Disease Control and Prevention, Nanjing, China; Department of Infectious Diseases, Nanjing Public Health Medical Center, the Second Hospital of Nanjing, Nanjing University of Chinese Medicine, Nanjing, China; Department of AIDS/STD Control and Prevention, Nanjing Municipal Central for Disease Control and Prevention, Nanjing, China; National Key Laboratory of Intelligent Tracking and Forecasting for Infectious Diseases, Chinese Center for Disease Control and Prevention, Beijing, China; Department of Infectious Diseases, Nanjing Public Health Medical Center, the Second Hospital of Nanjing, Nanjing University of Chinese Medicine, Nanjing, China; School of Public Health, Nanjing Medical University, Nanjing, China

**Keywords:** ART, CD4 recovery trajectory, HIV, piecewise linear mixed-effects model

## Abstract

**Background:**

Although CD4 recovery has been widely studied, its long-term temporal dynamics and phase-specific characteristics in the modern ART era remain incompletely characterized.

**Methods:**

This retrospective cohort study included adults with HIV in Nanjing, China, who initiated ART between 2010 and 2019 and maintained viral suppression. The optimal CD4 recovery trajectory model was identified using piecewise linear mixed-effects models with exhaustive grid search. Cumulative probability curves estimated probabilities of CD4 count recovery to ≥500 and ≥350 cells/μL.

**Results:**

2611 individuals contributing 22 970 person-years and 37 959 observations were analyzed. The best-fitting model identified a 4-phase CD4 recovery trajectory with breakpoints at 0.5, 2.5, and 6 years, characterized by the fastest increase during 0–0.5 years (265.4 cells/μL/year), progressive slowing during 0.5–2.5 and 2.5–6 years, and modest growth beyond 6 years (8.6 cells/μL/year). Cumulative probabilities of reaching both thresholds rose steadily but decelerated markedly after 6 years. When stratified by baseline CD4 counts at ART initiation, compared with the 350–499 subgroup, the <200 subgroup showed slower early CD4 count gains (≤49: −57.9; 50–199: −40.3 cells/μL/year) during 0–0.5 years, but accelerated increases during 0.5–2.5 years (≤49: +28.3; 50–199: +10.2 cells/μL/year) that persisted after 6 years (≤49: +4.7; 50–199: +5.3 cells/μL/year). Cumulative probabilities of reaching both thresholds in the <200 subgroup increased continuously throughout follow-up, whereas those with higher baseline levels plateaued at 6 years.

**Conclusions:**

CD4 recovery under sustained viral suppression followed a phase-specific trajectory. Individuals with advanced immunosuppression showed delayed but sustained CD4 recovery. These findings may help understand when CD4 recovery approaches its maximal potential.

Acquired immune deficiency syndrome (AIDS), caused by infection with HIV-1, remains a major global public health challenge. Although no effective HIV vaccine is currently available, the widespread use of ART enables durable suppression of viral replication and promotes CD4 count increase, resulting in substantial improvements in clinical outcomes and quality of life for people living with HIV (PLWH) [1–3]. Restoration of CD4 counts is a key indicator of immune reconstitution and is closely associated with the risk of AIDS-related events, non-AIDS comorbidities, and mortality [4–6].

Over the past 2 decades, CD4 recovery under suppressive ART has been widely studied. Previous studies have primarily focused on the magnitude and rate of CD4 recovery, the likelihood of achieving immunological normalization, and the factors associated with immune reconstitution [7–9]. Most studies have evaluated CD4 recovery at predefined time points or summarized long-term changes using average values. However, the long-term temporal dynamics of CD4 recovery remain incompletely characterized, particularly with respect to the existence and timing of a plateau phase. Some studies have reported a plateau in CD4 recovery after 2–5 years of ART [9–12], whereas others have documented continued increases in CD4 counts beyond this period [13, 14]. These inconsistencies may be related to limitations of earlier cohorts, including small sample sizes, short follow-up, and a low frequency of CD4 measurements. In addition, many of these studies were conducted in earlier treatment eras dominated by PI-based regimens and did not account for treatment modifications over time. Data from contemporary real-world cohorts with long-term follow-up may help further characterize the temporal dynamics of CD4 recovery under modern ART. A clearer understanding of the temporal dynamics of CD4 recovery may help clarify the timing of maximal CD4 recovery and thereby may inform appropriate timeframes for evaluating immune reconstitution efficacy, which vary greatly across studies [15–17].

Using a population-based cohort of PLWH with sustained viral suppression in Nanjing, China, we applied piecewise linear mixed-effects models combined with an exhaustive grid-search strategy to provide a data-driven characterization of long-term CD4 recovery trajectories and to identify potential breakpoints corresponding to phase transitions.

## METHODS

### Study Design and Participants

We conducted a population-based cohort study in eastern China, encompassing all 11 administrative districts of Nanjing (Xuanwu, Qinhuai, Jianye, Gulou, Pukou, Qixia, Yuhuatai, Jiangning, Liuhe, Lishui, and Gaochun). Individuals with HIV screening–positive results were referred to the Nanjing Municipal Centers for Disease Control and Prevention for confirmatory testing. Following laboratory confirmation of HIV infection, patients were referred to the only designated hospital for HIV/AIDS care (Nanjing Public Health Medical Center) for ART initiation and follow-up. More than 90% patients from all 11 municipal districts receive care at this center. This centralized diagnostic and treatment pathway enabled comprehensive population-level inclusion and longitudinal follow-up of individuals initiating ART.

The study included people with HIV aged 18 years or older who initiated ART between January 2010 and December 2019. The exclusion criteria were as follows: (1) follow-up duration <4 years; (2) 2 consecutive viral loads ≥200 copies/mL or a single viral load >1000 copies/mL after 48 weeks of ART; (3) absence of CD4 counts at ART initiation; (4) occurrence of malignancy, ART discontinuation, or death during the follow-up period. Follow-up commenced at ART initiation. Patients were followed monthly during the first 3 months and subsequently every 3 months at the outpatient clinic as part of routine clinical care. According to national guidelines, CD4 testing is provided free of charge once per year. In clinical practice, most patients undergo CD4 testing every 3–6 months, and at a minimum, at least 1 CD4 measurement per year is routinely performed. The endpoint of follow-up was August 2025. The study was designed according to the STROBE guidelines. The study was approved by the Ethics Committee of the Second Hospital of Nanjing (No. 2024-LS-ky076), and owing to the nature of the study, the requirement for informed consent was waived.

### Data Source

Demographic information, including gender, age, marital status, HIV transmission route, time of HIV diagnosis, was extracted from the China National HIV/AIDS Comprehensive Response Information Management System [18]. Clinical and laboratory data, including ART regimen, time of initiating ART, presence of opportunistic infections, HBV and HCV status, CD4 count and HIV viral load were extracted from the electronic medical record system of the Nanjing Public Health Medical Center.

### Statistical Analysis

To model the longitudinal trajectories of absolute CD4 count recovery, we employed piecewise linear mixed-effects models with random intercepts and random slopes. Analyses were based on CD4 values at their actual timing of each measurement, without annualization or averaging within calendar years.

Time since ART initiation was modeled using piecewise linear terms separated by 1 to 4 breakpoints, allowing the slope of CD4 recovery to vary across distinct recovery phases. For each specified number of breakpoints, we conducted an exhaustive grid search over all combinations of candidate locations. Candidate breakpoint locations were prespecified based on clinical plausibility and exploratory visualization, and were evaluated on a 0.5-year grid. Within each specified number of breakpoints, a separate model was fitted for each candidate combination, and the combination yielding the lowest Akaike Information Criterion (AIC) was selected. Based on prior clinical knowledge, we adjusted for a predefined set of covariates considered to potentially influence CD4 recovery in the final optimal model to estimate recovery slopes across phases. These covariates included age, gender, transmission route, CD4 counts at ART initiation, HCV infection, status of opportunistic infections, duration from HIV diagnosis to starting ART, and INSTI exposure during follow-up. INSTI exposure was treated as a time-dependent variable, indicating whether a patient was receiving an INSTI-based regimen at a given time point. INSTI exposure captured both INSTI-based regimens at ART initiation and switches to INSTI during follow-up.

From a clinical endpoint perspective, we estimated cumulative probability curves for CD4 recovery to thresholds of ≥500 and ≥350 cells/μL, defined as 2 consecutive measurements meeting each threshold, which are commonly used to evaluate immune reconstitution. Factors associated with time to CD4 recovery to both thresholds were examined using multivariable time-dependent Cox proportional hazards models that incorporated the INSTI exposure during follow-up as a time-dependent covariate.

To examine differences in CD4 recovery across baseline immune status at ART initiation, participants were stratified into 5 baseline CD4 subgroups: ≤49, 50–199, 200–349, 350–499, and ≥500 subgroups. Longitudinal changes in absolute CD4 counts were modeled using piecewise linear mixed-effects models with random intercepts and slopes. Time since ART initiation was represented by segmented linear terms separated by predefined breakpoints. Given prior evidence that INSTI-based therapies were associated with superior immune reconstitution [19], the model incorporated interaction terms between baseline CD4 subgroups and segmented time terms, as well as between the INSTI exposure and segmented time terms, with adjustment for other potential confounders. In addition, the cumulative probability curves for CD4 recovery to ≥350 and ≥500 cells/μL were also stratified by CD4 count at ART initiation.

Statistical significance was defined as a two-sided *P*-value <.05. All statistical analyses were performed using R software (version 4.4.0).

## RESULTS

### Characteristics of Study Participants

As shown in Supplementary Figure 1, a total of 4122 patients with HIV who initiated ART between 2010 and 2019 in Nanjing were initially screened. Patients were sequentially excluded due to follow-up <4 years (*N* = 501), missing viral load records (*N* = 31), virological failure after 48 weeks of ART (*N* = 380), ART discontinuation (*N* = 129), malignancy (*N* = 80), or death (*N* = 24) during follow-up, and absence of CD4 counts at ART initiation (*N* = 366). Finally, 2611 patients with sustained viral suppression were included, contributing to 22 970 person-years and 37 959 observations.

The median follow-up duration was 8.8 years (IQR, 6.8–10.4), with 31.4% (820/2611) followed for over 10 years. The median age was 33 years (IQR, 26–45), with most patients being male (93.3%), single (72.0%), and infected via MSM (74.7%). The median time from HIV diagnosis to ART initiation was 1.6 months (IQR, 0.9–3.2). The median CD4 counts at ART initiation were 281 cells/μL (IQR, 144–404 cells/μL). Over 90% of patients initiated ART with NNRTI-based regimens. During follow-up, 1123 patients (43.0%) were exposed to INSTI-based regimens, the majority of whom (*N* = 1029) switched to INSTI-based regimens after ART initiation. A minority had HBV, HCV, or opportunistic infections before ART. Participant characteristics are listed in Table 1.

**Table 1.** Characteristics of the Study Participants

Characteristic	Classification	Overall (*N* = 2611)	CD4 Counts At ART Initiation					*P*-Value
			≤49 (*N* = 335)	50–199 (*N* = 526)	200–349 (*N* = 847)	350–499 (*N* = 583)	≥500 (*N* = 320)	
Age at ART initiation (years)	—	33.0 (26.0–45.0)	39.0 (31.0–49.0)	37.0 (29.0–49.0)	33.0 (26.0–45.0)	29.0 (25.0–38.0)	29.0 (25.0–37.8)	<0.001
	≤30	1051 (40.3%)	70 (20.9%)	140 (26.6%)	361 (42.6%)	304 (52.1%)	176 (55.0%)	<0.001
	31–59	1439 (55.1%)	250 (74.6%)	351 (66.7%)	446 (52.7%)	258 (44.3%)	134 (41.9%)	
	≥60	121 (4.6%)	15 (4.5%)	35 (6.7%)	40 (4.7%)	21 (3.6%)	10 (3.1%)	
Gender	Male	2437 (93.3%)	319 (95.2%)	471 (89.5%)	785 (92.7%)	552 (94.7%)	310 (96.9%)	<0.001
	Female	174 (6.7%)	16 (4.8%)	55 (10.5%)	62 (7.3%)	31 (5.3%)	10 (3.1%)	…
Mode of transmission	MSM	1951 (74.7%)	205 (61.2%)	358 (68.1%)	656 (77.4%)	462 (79.3%)	270 (84.4%)	<0.001
	Heterosexual	621 (23.8%)	122 (36.4%)	152 (28.9%)	180 (21.3%)	119 (20.4%)	48 (15.0%)	
	Other	39 (1.5%)	8 (2.4%)	16 (3.0%)	11 (1.3%)	2 (0.3%)	2 (0.6%)	
Duration from diagnosis to ART initiation (months)	—	1.6 (0.9–3.2)	1.1 (0.7–2.1)	1.3 (0.7–2.9)	1.8 (1.0–3.7)	1.8 (1.1–3.3)	1.8 (1.1–4.6)	<0.001
	<1	730 (28.0%)	134 (40.0%)	190 (36.1%)	205 (24.2%)	132 (22.6%)	69 (21.6%)	<0.001
	≥1	1881 (72.0%)	201 (60.0%)	336 (63.9%)	642 (75.8%)	451 (77.4%)	251 (78.4%)	
Year of ART initiation	2010∼2014	792 (30.3%)	124 (37.0%)	160 (30.4%)	322 (38.0%)	120 (20.6%)	66 (20.6%)	<0.001
	2015∼2019	1819 (69.7%)	211 (63.0%)	366 (69.6%)	525 (62.0%)	463 (79.4%)	254 (79.4%)	…
Follow-up from ART initiation (years)	—	8.8 (6.8–10.4)	9.2 (7.0–11.4)	8.6 (6.7–10.5)	9.2 (7.0–11.1)	8.5 (6.7–10.0)	8.1 (6.4–9.7)	<0.001
Baseline CD4 counts (cells/μL)	—	281.0 (144.0–404.0)	17.0 (8.0–31.0)	126.0 (84.0–168.0)	278.0 (243.0–312.0)	413.0 (380.0–459.0)	597.0 (534.3–706.0)	<0.001
Baseline ART regimen	NNRTI-based	2467 (94.5%)	294 (87.7%)	502 (95.4%)	816 (96.3%)	556 (95.4%)	299 (93.4%)	<0.001
	INSTI-based	94 (3.6%)	19 (5.7%)	15 (2.9%)	25 (3.0%)	18 (3.1%)	17 (5.3%)	
	PI-based	50 (1.9%)	22 (6.6%)	9 (1.7%)	6 (0.7%)	9 (1.5%)	4 (1.3%)	
INSTI exposure	Ever received INSTI	1123 (43.0%)	141 (42.1%)	200 (38.0%)	373 (44.0%)	254 (43.6%)	155 (48.4%)	0.045
	Never received INSTI	1488 (57.0%)	194 (57.9%)	326 (62.0%)	474 (56.0%)	329 (56.4%)	165 (51.6%)	
HBV infection	Yes	198 (7.6%)	36 (10.8%)	41 (7.8%)	68 (8.0%)	38 (6.5%)	15 (4.7%)	0.043
	No	2413 (92.4%)	299 (89.2%)	485 (92.2%)	779 (92.0%)	545 (93.5%)	305 (95.3%)	
HCV infection	Yes	27 (1.0%)	4 (1.2%)	11 (2.1%)	7 (0.8%)	2 (0.3%)	3 (0.9%)	0.068
	No	2584 (99.0%)	331 (98.8%)	515 (98.0%)	840 (99.2%)	581 (99.7%)	317 (99.1%)	
Opportunistic infection	Yes	329 (12.6%)	209 (62.4%)	95 (18.1%)	19 (2.2%)	5 (0.9%)	1 (0.3%)	<0.001
	No	2282 (87.4%)	126 (37.6%)	431 (81.9%)	828 (97.8%)	578 (99.1%)	319 (99.7%)	

Ever received INSTI includes both patients initiating ART with INSTI-based regimens and those who switched to INSTI-based regimens during follow-up.

### Model Selection and Identification of CD4 Recovery Breakpoints

Under the settings of 1 to 4 breakpoints, multiple piecewise linear mixed-effects models were fitted for each setting, and the model with the minimum AIC was selected. The corresponding AIC values of these best-fitting models were 476,617, 475,975, 475,908, and 475,904, respectively (Supplementary Table 1). The corresponding optimal breakpoint locations after ART initiation were 1 year; 0.5 and 4 years; 0.5, 2.5, and 6 years; and 0.5, 2.5, 6, and 12 years. Although model fit improved progressively with the inclusion of additional breakpoints, the 3-breakpoint model was selected as the optimal model for balancing fit and parsimony because the improvement in AIC from 3 to 4 breakpoints was minimal.

### Trajectory Analysis of Absolute CD4 Count Increase

**Figure 1.**
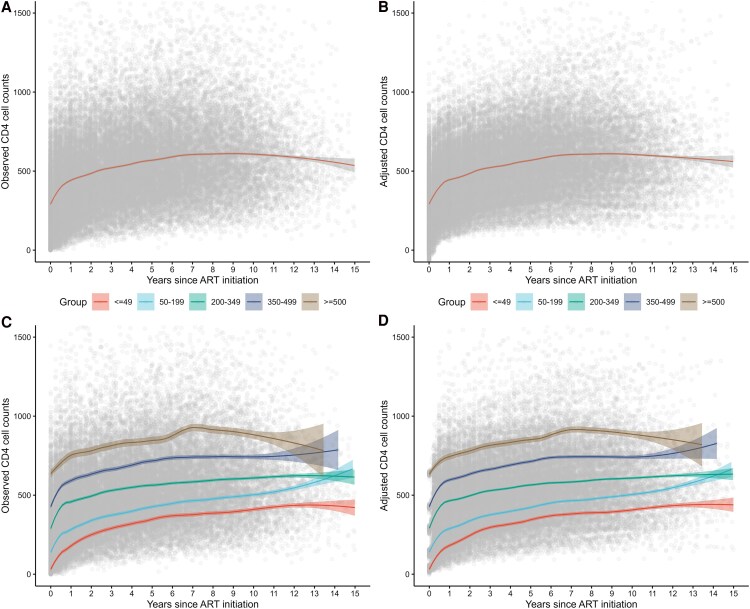
Trajectory analysis of absolute CD4 count increase. *A* and *B*, Observed and adjusted trajectories of absolute CD4 count increase. The adjusted trajectories represent the model-predicted values from piecewise linear mixed-effects models across intervals of 0–0.5 y, 0.5–2.5 y, 2.5–6 y, and beyond 6 y, adjusted for age, gender, transmission route, CD4 count at ART initiation, HCV infection, opportunistic infections, year of ART initiation, duration from HIV diagnosis to starting ART, and INSTI exposure. Lines represent observed and predicted mean values. Shaded areas indicate 95% CI, and dots represent individual observations. *C* and *D*, Observed and adjusted trajectories of absolute CD4 counts increase across baseline CD4 subgroups. The adjusted trajectories represent the model-predicted values from piecewise linear mixed-effects models across 4-time intervals within baseline CD4 subgroups, adjusted for age, gender, marital status, transmission route, opportunistic infections, year of ART initiation, duration from HIV diagnosis to starting ART, and INSTI exposure. Lines represent observed and predicted mean values. Shaded areas indicate 95% CI, and dots represent individual observations.

**Figure 2.**
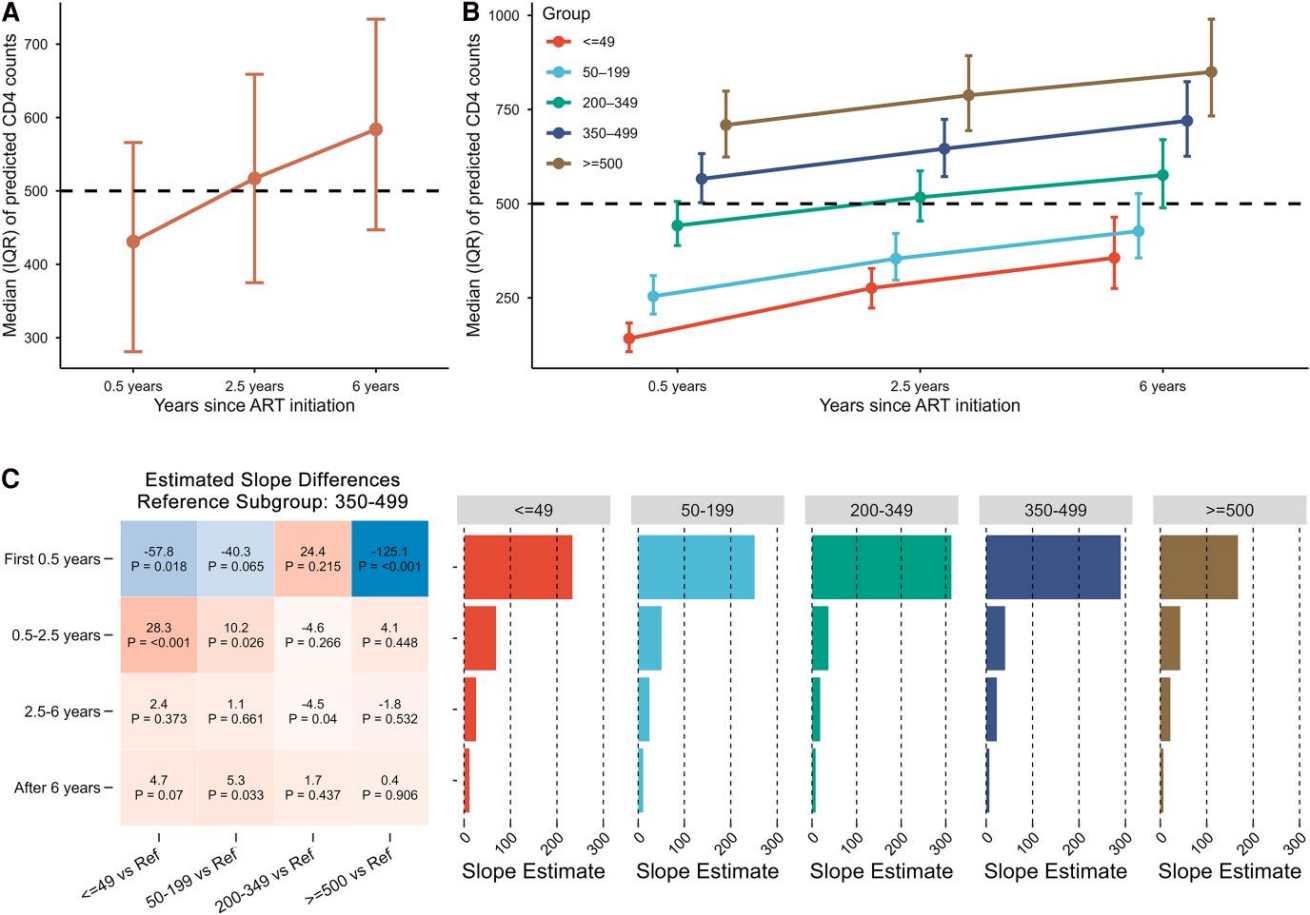
Phase-specific CD4 recovery trajectories stratified by CD4 count at ART initiation. *A*, Median (IQR) of model-predicted CD4 counts at the 3 breakpoints. Time points correspond to 0.5, 2.5, and 6 y after ART initiation. Points represent median values and error bars denote interquartile ranges. *B*, Median (IQR) of model-predicted CD4 counts at the 3 breakpoints across 5 baseline CD4 subgroups. Time points correspond to 0.5, 2.5, and 6 y after ART initiation. Points represent median values and error bars denote interquartile ranges. *C*, Phase-specific differences in CD4 recovery slopes stratified by CD4 counts at ART initiation. Left panel shows differences in estimated CD4 count recovery slopes compared with the 350–499 subgroup. Values represent the difference in slope estimates, with corresponding *P*-values indicated in each cell. Right panels display the estimated slopes for each baseline CD4 subgroup (≤49, 50–199, 200–349, 350–499, and ≥500 subgroup) across the 4-time intervals.

Distinct phases of absolute CD4 count increase were evident. The observed (raw) and model-predicted (adjusted) trajectories are shown in Figure 1*A* and *B*, respectively. The estimated slope of CD4 count increase was highest in the initial phase, reaching 265.4 cells/μL/year (95% CI, 252.0–278.7) in the first 0.5 years. It subsequently declined to 46.3 cells/μL/year (95% CI, 43.1–49.4) between 0.5 and 2.5 years, and further to 21.7 cells/μL/year (95% CI, 19.9–23.5) between 2.5 and 6 years. Beyond 6 years, CD4 cell counts continued to rise, but the annual gain was modest, averaging only 8.63 cells/μL/year (95% CI, 6.7–10.6) (Table 2). The median and IQR of model-predicted CD4 counts at the 3 breakpoints were 431 (281–566) cells/μL, 517 (375–659) cells/μL, and 584 (447–734) cells/μL, respectively (Figure 2*A*;  Supplementary Table 2).

**Table 2.** Estimated Slopes of CD4 Recovery Across Four Time Periods

Breakpoints	Estimated Slopes (cells/μL/year), 95% CI	*P*-Value
Total patients		
0–0.5 y	265.4 (252.0–278.7)	<0.001
0.5–2.5 y	46.3 (43.1–49.4)	<0.001
2.5–6 y	21.7 (19.9–23.5)	<0.001
>6 y	8.6 (6.7–10.6)	<0.001
CD4 counts at ART initiation ≤49 cells/μL		
0–0.5 y	233.5 (209.5–257.5)	<0.001
0.5–2.5 y	68.6 (62.7–74.5)	<0.001
2.5–6 y	25.7 (21.8–29.6)	<0.001
>6 y	11.6 (7.6–15.7)	<0.001
CD4 counts at ART initiation 50–199 cells/μL		
0–0.5 y	251.5 (228.3–274.8)	<0.001
0.5–2.5 y	51.1 (45.6–56.6)	<0.001
2.5–6 y	24.3 (21.0–27.6)	<0.001
>6 y	10.6 (7.0–14.1)	<0.001
CD4 counts at ART initiation 200–349 cells/μL		
0–0.5 y	312.4 (289.7–335.1)	<0.001
0.5–2.5 y	36.9 (31.6–42.2)	<0.001
2.5–6 y	18.3 (15.4–21.3)	<0.001
>6 y	8.1 (5.0–11.1)	<0.001
CD4 counts at ART initiation 350–499 cells/μL		
0–0.5 y	289.6 (256.1–323.2)	<0.001
0.5–2.5 y	40.8 (32.8–48.8)	<0.001
2.5–6 y	22.9 (18.4–27.4)	<0.001
>6 y	6.9 (1.5–12.3)	0.006
CD4 counts at ART initiation ≥500 cells/μL		
0–0.5 y	167.1 (112.1–222.1)	<0.001
0.5–2.5 y	42.8 (29.5–56.1)	<0.001
2.5–6 y	21.8 (13.8–29.7)	<0.001
>6 y	6.7 (−2.9–16.3)	0.278

*P*-values represent tests of whether the estimated CD4 slope in each time period differs from zero.

### Trajectory Analysis of Absolute CD4 Count Increase Stratified by CD4 Count at ART Initiation

Patients were stratified by CD4 counts at ART initiation into 5 subgroups (≤49, 50–199, 200–349, 350–499, and ≥500 subgroups). Across all subgroups, absolute CD4 count recovery followed a consistent 4-phase trajectory characterized by rapid gains within the first 0.5 years, a slower increase between 0.5 and 2.5 years, further attenuation from 2.5 to 6 years, and minimal gains thereafter (Figure 1*C* and *D*; Table 2).

Patients with CD4 counts at ART initiation ≥500 cells/μL showed no significant increase beyond 6 years (*P* = .278), reflecting a ceiling effect. The model-predicted CD4 counts at the 3 breakpoints across subgroups were shown in Figure 2*B*. Exposure to INSTI tended to be associated with a larger CD4 recovery slope during 0–0.5 years after ART initiation (+41.8 cells/μL per year; *P* = .180) but a smaller slope during 0.5–2.5 years (−13.6 cells/μL per year; *P* = .035), with no material difference thereafter (Supplementary Table 3).

After controlling for the time-varying covariate of INSTI exposure, we compared the differences in the slopes of CD4 count increase among different subgroups. The 350–499 subgroup was chosen as the reference, as it represented individuals with relatively preserved immune function and showed a typical CD4 recovery trajectory. Compared with the reference, distinct features emerged across strata (Figure 2*C*; Supplementary Table 3). First, the ≥500 subgroup had a significantly lower early-phase slope during the first 0.5 years (−125.1 cells/μL/year; *P* < .001), consistent with a ceiling effect in individuals close to normal baseline levels. Second, the 200–349 subgroup showed recovery slopes comparable to the reference throughout follow-up. Third, the subgroups of 50–199 and ≤49 had slower early increase (50–199 subgroup: −40.3 cells/μL/year, *P* = .065; ≤49 subgroup: −57.9 cells/μL/year, *P* = .018) during the first 0.5 years. However, both subgroups accelerated thereafter, with slopes exceeding the reference during 0.5–2.5 years (50–199 subgroup: +10.2 cells/μL/year, *P* = .026; ≤49 subgroup: +28.3 cells/μL/year, *P* < .001). After 6 years, both subgroups modestly exceeded the reference (50–199 subgroup: +5.3 cells/μL/year; *P* = .033; ≤49 subgroup: +4.7 cells/μL/year; *P* = .070). These findings revealed the “catch-up” recovery in absolute CD4 counts among patients with CD4 counts at ART initiation <200 cells/μL.

### Cumulative Probability of CD4 Recovery to ≥500 and ≥350 Cells/μL

**Figure 3.**
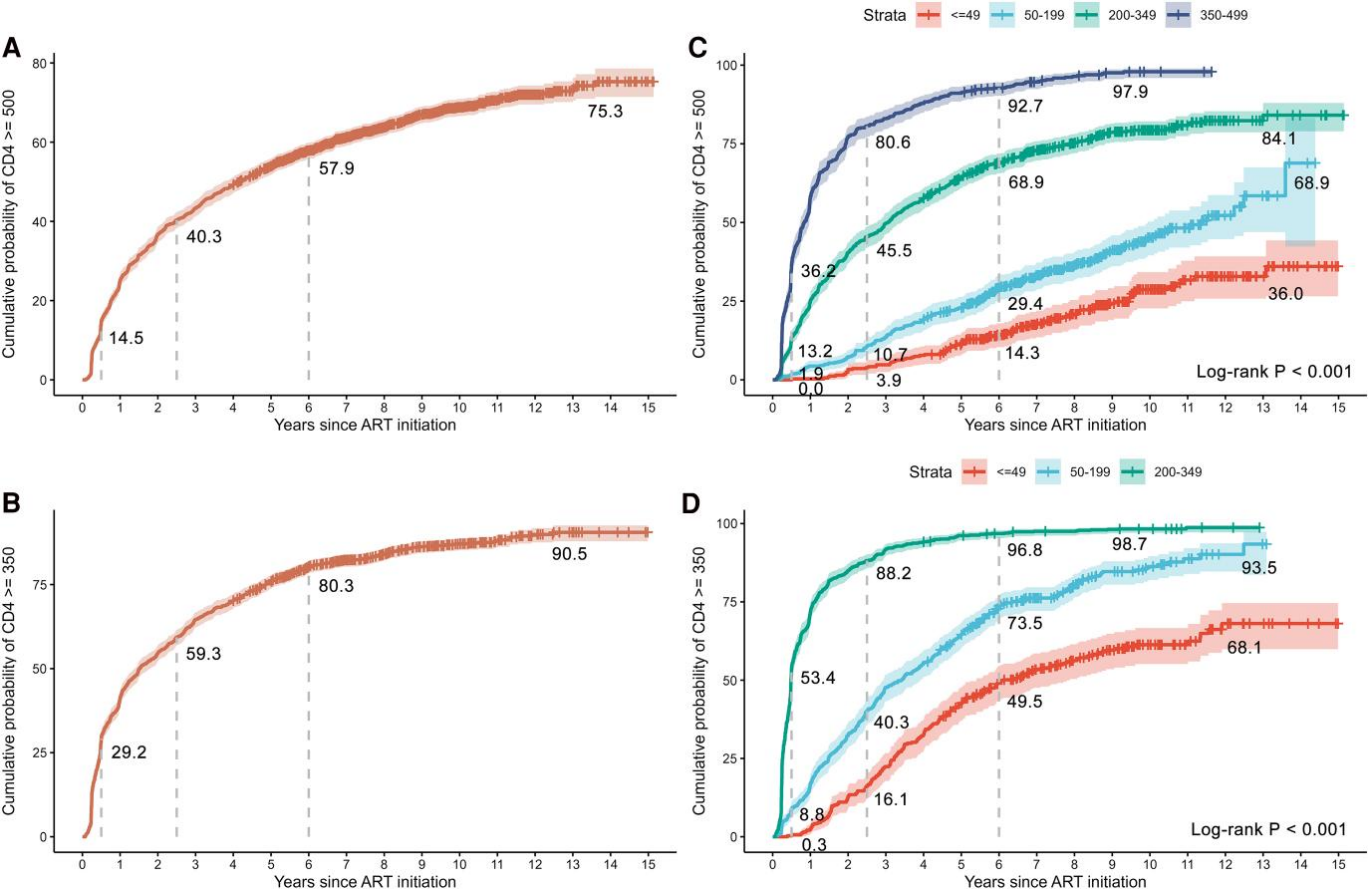
Cumulative probability of CD4 recovery to ≥500 and ≥350 cells/μL. *A* and *B*, The cumulative probability curves of reaching CD4 counts ≥500 and ≥350 cells/μL among patients with CD4 count at ART initiation below the corresponding thresholds. *C* and *D*, The cumulative probability curves of reaching CD4 counts ≥500 and ≥350 cells/μL across baseline CD4 subgroups.

We evaluated the cumulative probability of CD4 recovery to ≥500 and ≥350 cells/μL among patients with CD4 counts at ART initiation below the corresponding thresholds. Overall, the cumulative probability of reaching both thresholds increased rapidly within the first 0.5 years, coinciding with the phase of the most pronounced increase in absolute CD4 counts (Figure 3*A* and *B*). During this early period, the cumulative probability increased by an average of 29.1% per year for recovery to ≥500 cells/μL and 58.4% per year for recovery to ≥350 cells/μL. By year 6, the cumulative probability reached 57.9% for recovery to ≥500 cells/μL and 80.3% for recovery to ≥350 cells/μL. During extended follow-up, the curves increased modestly, with an additional 17.4% (1.9% per year) for recovery to ≥500 cells/μL and 10.2% (1.1% per year) for recovery to ≥350 cells/μL. These findings indicate that cumulative probability of achieving both CD4 thresholds increased continuously, with the most rapid gains occurring within the first 6 years and a marked deceleration thereafter.

### Cumulative Probability of CD4 Recovery to ≥500 and ≥350 Cells/μL Stratified by CD4 Count at ART Initiation

When stratified by CD4 count at ART initiation, distinct subgroup-specific patterns also emerged (Figure 3*C* and *D*). Using the ≥500 cells/μL threshold, patients with CD4 counts ≥200 cells/μL at ART initiation exhibited rapid increases in cumulative probability within the first 0.5 years, followed by early plateauing by around year 6. Thereafter, gains in cumulative probability were minimal, with average annual gains of 1.7% in the 200–349 subgroup and 0.9% in the 350–499 subgroup. In contrast, patients with CD4 counts at ART initiation <200 cells/μL showed slower early increases during the first 0.5 years but continued to increase steadily thereafter, without reaching a plateau by year 6. By the end of follow-up, cumulative probabilities reached 68.9% in the 50–199 subgroup and 36.0% in the ≤49 subgroup (Figure 3*C*). Using the ≥350 cells/μL threshold, patients with CD4 counts at ART initiation <200 cells/μL similarly demonstrated persistent increases in cumulative probability without plateauing by year 6, with cumulative probabilities reaching 93.5% in the 50–199 subgroup and 68.1% in the ≤49 subgroup at the end of follow-up (Figure 3*D*). These findings indicated that, among patients with CD4 counts at ART initiation <200 cells/μL, the continuous increases in cumulative probability of reaching both thresholds were consistent with the “catch-up” recovery of absolute CD4 counts.

### Factors Associated With CD4 Recovery to ≥500 and ≥350 Cells/μL

In multivariable time-dependent Cox models, CD4 count at ART initiation was the strongest determinant of CD4 recovery to both thresholds. For recovery to ≥500 cells/μL (reference: the 350–499 subgroup), the adjusted hazard ratios (aHRs) were 0.08 (95% CI, .06–.10; *P* < .001) for the ≤49 subgroup, 0.13 (95% CI, .11–.15; *P* < .001) for the 50–199 subgroup, and 0.38 (95% CI, .34–.44; *P* < .001) for the 200–349 subgroup (Supplementary Figure 2A). For recovery to ≥350 cells/μL (reference: the 200–349 subgroup), the aHRs were 0.15 (95% CI, .13–.19; *P* < .001) for the ≤49 subgroup and 0.28 (95% CI, .24–.32; *P* < .001) for the 50–199 subgroup (Supplementary Figure 2B). In addition, older age and a history of opportunistic infection were associated with a lower likelihood of reaching both CD4 thresholds (Supplementary Figures 2A and B). Notably, INSTI exposure was independently associated with a higher likelihood of CD4 recovery to ≥350 cells/μL (aHR = 1.48; 95% CI, 1.21–1.80; *P* < .001) (Supplementary Figure 2B).

## DISCUSSION

In this study, we constructed the phase-specific trajectory of CD4 recovery using a data-driven modeling approach based on a large real-world Chinese cohort. The optimal piecewise linear mixed-effects model identified 3 breakpoints at 0.5, 2.5, and 6 years after ART initiation, thereby dividing the CD4 recovery trajectory into 4 phases: a rapid initial increase within the first 0.5 years, a slower rise from 0.5 to 2.5 years, further attenuated growth between 2.5 and 6 years, and modest continued increases beyond 6 years. In contrast to earlier studies that reported a plateau after several years of therapy [9–11, 20], we observed continued increases in CD4 counts throughout follow-up in our cohort, with a corresponding rise in the cumulative probability of reaching key CD4 thresholds (350 and 500 cells/μL). Our findings support the notion that maintaining ART confers ongoing benefits for the restoration of the immune system [21, 22].

When stratified by CD4 counts at ART initiation, a consistent 4-phase recovery pattern was observed across all strata, although the magnitude of recovery differed signiﬁcantly between subgroups. Patients with baseline CD4 counts <200 cells/μL exhibited slower early recovery but a subsequent “catch-up” growth pattern that extended beyond 6 years. Notably, with the exception of the baseline CD4 ≥ 500 subgroup, all other subgroups continued to show increases in absolute CD4 counts beyond 6 years. However, the clinical relevance of this late-phase CD4 counts increase varied according to baseline immune status. Among patients with CD4 counts at ART initiation <200 cells/μL, late-phase gains in CD4 counts translated into continuously reaching key CD4 thresholds. By contrast, among those with relatively preserved baseline immunity, late-phase increases were not accompanied by additional achievement of CD4 thresholds, as most had already reached these thresholds by around 6 years of ART. These results may explain why no plateau was observed in the cumulative probability curves of thresholds attainment among patients with baseline CD4 counts <200 cells/μL, whereas cumulative probabilities stabilized around 6 years among those with higher CD4 counts at ART initiation. Although patients with CD4 counts at ART initiation <200 cells/μL had sustained CD4 recovery during follow-up, the proportion achieving the key threshold of 500 cells/μL—reflecting functional normalization of CD4 counts—remained limited, particularly in the ≤49 subgroup, in which only one-third of patients ultimately reached this level. Due to the nature of the retrospective study, these estimates may only serve as descriptive reference information to support clinical counseling and expectation management, rather than as tools for individual-level prediction. Our findings reinforce the importance of early ART initiation before the development of severe immunodeficiency [23–26].

From a biological perspective, CD4 recovery after ART is also a stage-dependent process, with different mechanisms predominating at different phases. Early increases are largely driven by redistribution of memory CD4^+^ T cells following viral suppression and reduced immune activation, resulting in a rapid rise in circulating CD4 counts [15, 27].

Subsequent recovery gradually slows and is mainly sustained by thymic output and naïve T-cell generation [28], followed by more modest increases at later stages that are largely attributable to homeostatic proliferation and prolonged cell survival [29]. In individuals with advanced baseline immunosuppression, early recovery may be constrained by severe immune damage and lymphoid tissue disruption, whereas later improvements may reflect gradual restoration through homeostatic mechanisms. The phase-specific structure identified by our data-driven model provides a temporal framework that may help delineate these biological stages and their transitions, and may facilitate future studies investigating the mechanisms of incomplete CD4 recovery.

In China, INSTIs have not yet been included in the first-line regimens of the national free antiretroviral therapy program. Consequently, most patients initiated ART with NNRTI-based regimens. Due to the superior efficacy, safety, and tolerability of INSTI in clinical practice [30, 31], 39.41% (*N* = 1029) of patients have switched to INSTI-based therapy during follow-up. To account for potential confounding introduced by treatment switching, INSTI exposure during follow-up was modeled as a time-varying covariate across all analyses. We observed an early benefit of INSTI on increases in absolute CD4 counts. Consistently, in time-to-event analyses, INSTI use was also associated with higher probabilities of CD4 recovery to key thresholds. These findings are consistent with results from the CoRIS cohort [19], which demonstrated an early immunological advantage associated with INSTI use. The accelerated immunological restoration may be attributable to more rapid viral suppression and earlier immune quiescence with INSTI-based regimens [19, 32–34]. However, as patients receiving non-INSTI-based regimens achieved sustained viral suppression, differences in CD4 recovery between treatment regimens gradually diminished over time. This observation aligns with previous studies indicating that switching ART in virally suppressed patients does not significantly affect CD4 recovery [35, 36].

Our trajectory-based findings may help improve understanding of when CD4 recovery approaches its maximal potential. This information may help inform the selection of appropriate timeframes for the assessment of immune reconstitution efficacy, particularly immunological non-response (INR), which is typically evaluated by CD4 counts or increase slopes under sustained virological suppression. Several reviews have highlighted substantial heterogeneity in the time points used to assess INR across studies [15–17]. The data-driven temporal patterns identified in this study may therefore provide useful reference information for this issue. Under sustained virological suppression, for individuals with higher CD4 counts at ART initiation, a duration of ∼6 years of ART may serve as a reasonable reference for evaluating immune reconstitution efficacy. In contrast, no clear plateau in CD4 recovery was observed among patients with baseline immunosuppression, as indicated by continued increases in CD4 counts and steadily rising cumulative probabilities of reaching key CD4 thresholds. These findings suggest that a longer evaluation period may be required in this population.

This study has several limitations. First, patients with short follow-up, virological failure, ART discontinuation, malignancy, or death were excluded to enable modeling of long-term CD4 recovery trajectories under sustained virological suppression. Individuals excluded by these criteria may differ systematically from those included in the analysis. This selection strategy may introduce selection bias, resulting in an analytic population enriched for individuals with more stable clinical conditions, which may limit the generalizability of our findings to broader clinical populations. Furthermore, transient viral blips and low-level viremia were not specifically evaluated in this study. These viral dynamics may also influence immune recovery, and future studies incorporating more detailed viral load dynamics are needed to further clarify their impact. Second, immune reconstitution is a multidimensional process. In addition to CD4 counts, CD8 counts and the CD4/CD8 ratio have been increasingly recognized as important indicators of immune reconstitution in the modern ART era. Future studies integrating these markers are needed to provide a more comprehensive evaluation of immune reconstitution. Third, the number of individuals with very long-term follow-up was relatively limited in the later years of care. Consequently, data density beyond 10 years after ART initiation was lower than in earlier periods. This reduced sample size may lead to less stable trajectory estimates and more uncertainty in the late follow-up period. Therefore, the trajectory patterns observed during very long-term follow-up should be interpreted with caution. Finally, as free viral load testing when initiating ART was not routinely available in China before the 2023 national policy, viral load data at ART initiation were missing for many patients. Therefore, we were unable to adjust for viral load at ART initiation in our analyses, and its potential impact on CD4 recovery needs further evaluation.

## CONCLUSIONS

In this study, data-driven piecewise modeling identified a phase-specific trajectory of CD4 recovery, with breakpoints at 0.5, 2.5, and 6 years after ART initiation. Individuals with advanced baseline immunosuppression exhibited delayed but sustained CD4 recovery over time. These findings may help understand the timing of maximal CD4 recovery, and thereby help inform appropriate timeframes for evaluating immune reconstitution efficacy.

## Supplementary Material

ofag181_Supplementary_Data

## Supplementary Data

Supplementary materials are available at *Open Forum Infectious Diseases* online. Consisting of data provided by the authors to benefit the reader, the posted materials are not copyedited and are the sole responsibility of the authors, so questions or comments should be addressed to the corresponding author.
