# Supplementary material for: Data-driven Modeling of Long-term CD4 Cell Recovery Trajectories Under Modern Antiretroviral Therapy in People Living With HIV
Source: Open Forum Infect Dis. 2026 Mar 27;13(4):ofag181. doi: 10.1093/ofid/ofag181 (PMC13061125; doi:10.1093/ofid/ofag181)
Supplement: ofag181_Supplementary_Data [file ofag181_supplementary_data.docx]

**Supplementary Materials**

**Data-driven modelling of long-term CD4 cell recovery trajectories under modern antiretroviral therapy in people living with HIV**

**The file includes:**

Description of supplemental tables and figures

Supplementary Figure S1 to S2

Supplementary Tables S1 to S3

**Description of supplemental tables and figures**

**Figure S1.** The flow chart of sample selection in this study.

**Figure S2.** Time-dependent Cox regression analysis of factors associated with CD4 recovery to ≥500 and ≥350 cells/μL.

**Table S1.** The model with the minimum AIC under the settings of 1 to 4 breakpoints.

**Table S2.** Predicted CD4 counts at three breakpoints.

**Table S3.** Group differences in estimated slope across four time periods

**Figure S1.** The flow chart of sample selection in this study.


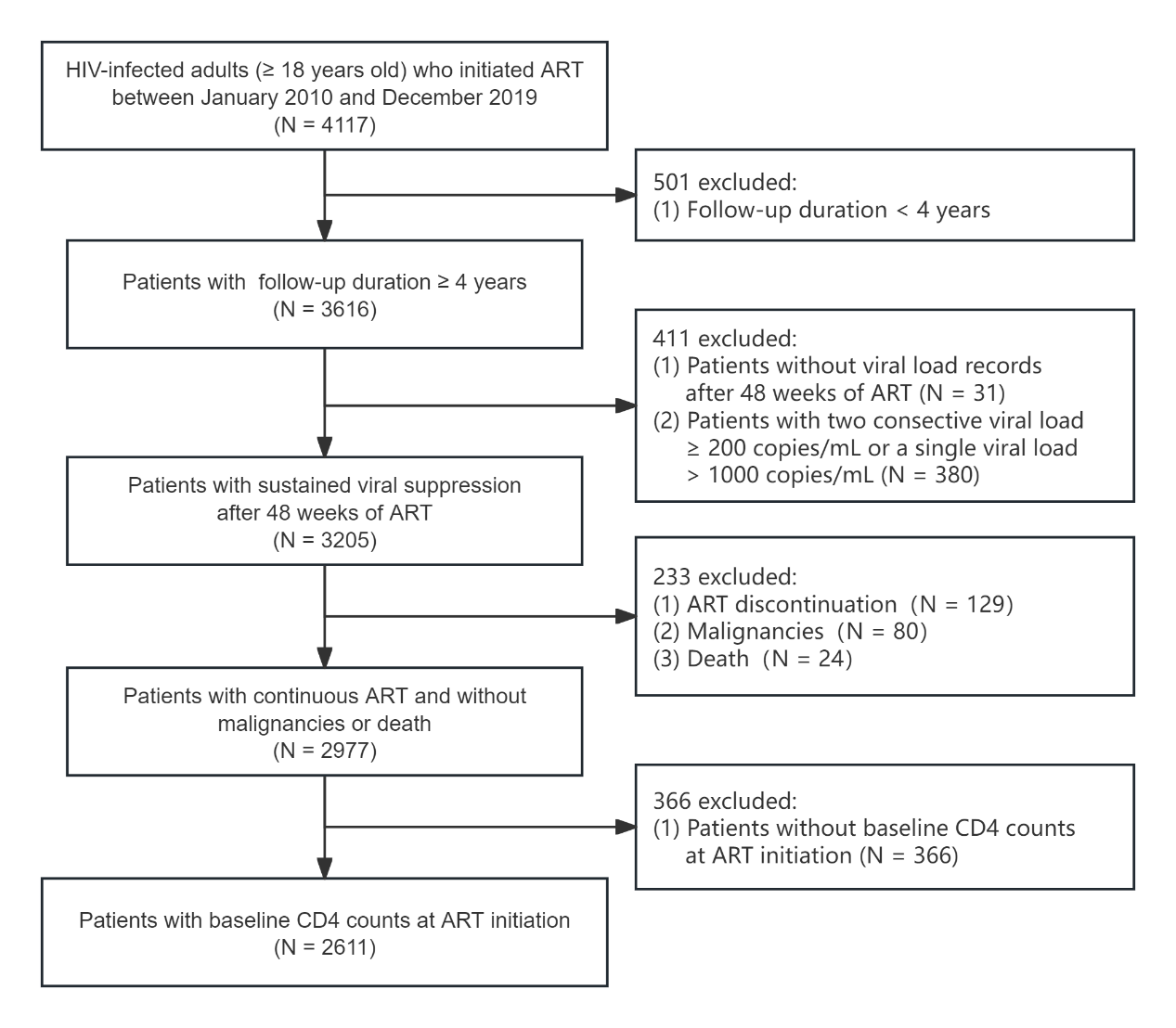


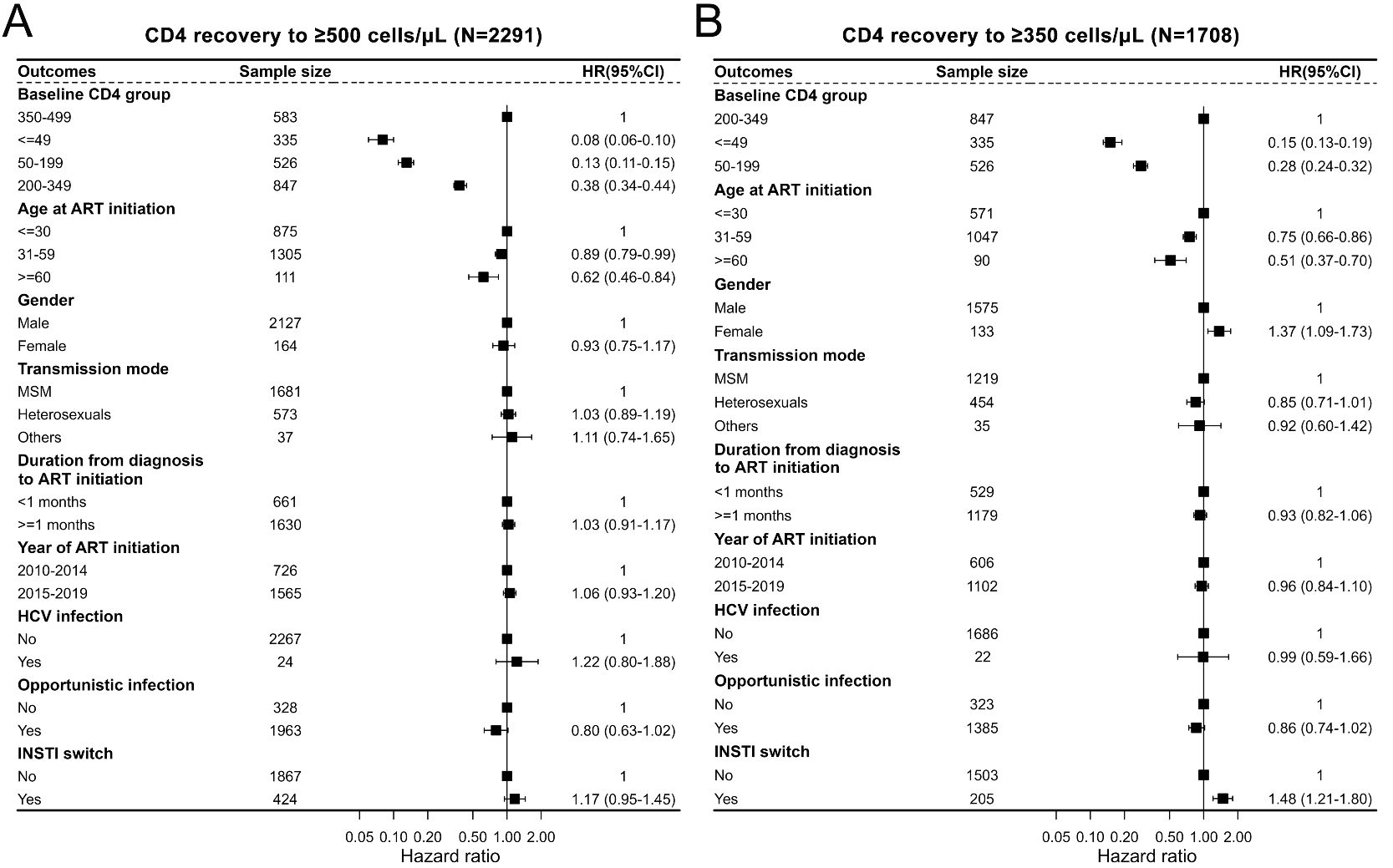
**Figure S2. Time-dependent Cox regression analysis of factors associated with CD4 recovery to ≥500 and ≥350 cells/μL.** Forest plots show adjusted hazard ratios (HRs) with 95% CI for factors associated with CD4 recovery to ≥500 cells/μL **(A)** and ≥350 cells/μL **(B)**.

**Table S1. The model with the minimum AIC under the settings of one to four breakpoints.**

| Number of breakpoints | Breakpoints location (Years after ART) | AIC value |
| --- | --- | --- |
| 1 | 1 | 476,617 |
| 2 | 0.5 | 475,975 |
|  | 3.5 |  |
| 3 | 0.5 | 475,908 |
|  | 2.5 |  |
|  | 6 |  |
| 4 | 0.5 | 475,904 |
|  | 2.5 |  |
|  | 6 |  |
|  | 12 |  |

**Table S2. Median (IQR) of predicted CD4 counts at the three breakpoints**

| Breakpoints | Median, IQR (cells/μL) |
| --- | --- |
| **Total patients** |  |
| 0.5 years | 431 (281–566) |
| 2.5 years | 517 (375–659) |
| 6 years | 584 (447–734) |
| **Baseline CD4 counts ≤49 cells/μL** |  |
| 0.5 years | 142 (107–183) |
| 2.5 years | 276 (223–328) |
| 6 years | 356 (275–464) |
| **Baseline CD4 counts 50-199 cells/μL** |  |
| 0.5 years | 254 (207–309) |
| 2.5 years | 354 (297–421) |
| 6 years | 427 (356–527) |
| **Baseline CD4 counts 200-349 cells/μL** |  |
| 0.5 years | 442 (389–506) |
| 2.5 years | 517 (454–587) |
| 6 years | 576 (489–670) |
| **Baseline CD4 counts 350-499 cells/μL** |  |
| 0.5 years | 566 (503–633) |
| 2.5 years | 646 (572–724) |
| 6 years | 720 (626–824) |
| **Baseline CD4 counts ≥500 cells/μL** |  |
| 0.5 years | 709 (624–799) |
| 2.5 years | 788 (694–893) |
| 6 years | 850 (733–990) |

**Table S3. Group differences in estimated slope across four time periods**

| Comparison | β (95%CI) | ^a^P value |
| --- | --- | --- |
| **0-0.5 years** |  |  |
| **Reference (350-499)** | ––– | ––– |
| ≤49*Time (test for interaction) | -57.8 (-105.9 to -9.8) | 0.018 |
| 50-199*Time (test for interaction) | -40.3 (-83.1 to 2.5) | 0.065 |
| 200-349*Time (test for interaction) | 24.4 (-14.2 to 62.9) | 0.215 |
| ≥500*Time (test for interaction) | -125.1 (-175.1 to -75.1) | <0.001 |
| **Reference (Never INSTI exposure)** | ––– | ––– |
| Ever INSTI exposure *Time (test for interaction) | 41.8 (-19.5 to 103.03) | 0.180 |
| **0.5-2.5 years** |  |  |
| **Reference (350-499)** | ––– | ––– |
| ≤49*Time (test for interaction) | 28.3 (18.3 to 38.2) | <0.001 |
| 50-199*Time (test for interaction) | 10.2 (1.2 to 19.1) | 0.026 |
| 200-349*Time (test for interaction) | -4.6 (-12.7 to 3.5) | 0.266 |
| ≥500*Time (test for interaction) | 4.1 (-6.5 to 14.7) | 0.448 |
| **Reference (Never INSTI exposure)** | ––– | ––– |
| Ever INSTI exposure *Time (test for interaction) | -13.6 (-26.2 to -0.92) | 0.035 |
| **2.5-6 years** |  |  |
| **Reference (350-499)** | ––– | ––– |
| ≤49*Time (test for interaction) | 2.4 (-2.9 to 7.7) | 0.373 |
| 50-199*Time (test for interaction) | 1.1 (-3.7 to 5.8) | 0.661 |
| 200-349*Time (test for interaction) | -4.5 (-8.8 to -0.2) | 0.040 |
| ≥500*Time (test for interaction) | -1.8 (-7.4 to 3.8) | 0.532 |
| **Reference (Never INSTI exposure)** | ––– | ––– |
| Ever INSTI exposure *Time (test for interaction) | -0.2 (-4.8 to 4.4) | 0.922 |
| **After 6 years** |  |  |
| **Reference (350-499)** | ––– | ––– |
| ≤49*Time (test for interaction) | 4.7 (-0.4 to 9.8) | 0.070 |
| 50-199*Time (test for interaction) | 5.3 (0.4 to 10.1) | 0.033 |
| 200-349*Time (test for interaction) | 1.7 (-2.6 to 6.0) | 0.437 |
| ≥500*Time (test for interaction) | 0.4 (-5.7 to 6.4) | 0.906 |
| **Reference (Never INSTI exposure)** | ––– | ––– |
| Ever INSTI exposure *Time (test for interaction) | -0.61 (-3.3 to 2.1) | 0.659 |

Note: ^a^P value represents the significance of the interaction term, adjusted for age, gender, transmission route, opportunistic infections, HCV infections, year of ART initiation, and duration from HIV diagnosis to starting ART.
